# Genome-Wide Identification and Expression Analysis of the TIM Gene Family in Sea-Island Cotton (*Gossypium barbadense*) During Fiber Development

**DOI:** 10.3390/cimb48090929

**Published:** 2026-09-11

**Authors:** Zixin Zhou, Weiran Wang, Meng Wang, Caixia Li, Yaohua Li, Lingfang Ran, Jianping Li, Zhiqing Liu, Jiahui Zhu, Jing Yang, Yifan Wang, Yahui Deng, Wumaierjiang Kuerban, Alifu Aierxi, Jie Kong, Nan Zhao

**Affiliations:** Xinjiang Key Laboratory of Cotton Genetic Improvement and Intelligent Production, Cotton Research Institute of Academy of Agricultural Sciences of Xinjiang Uyghur Autonomous Region, Urumqi 830091, China; zhouzixin2023@xaas.ac.cn (Z.Z.); wangweiran2011@xaas.ac.cn (W.W.); wangmeng2021@xaas.ac.cn (M.W.); licaixia0102@163.com (C.L.); liyaohua2023@xaas.ac.cn (Y.L.); 17623314900@163.com (L.R.); ljp7786@xaas.ac.cn (J.L.); lzq9102@163.com (Z.L.); zhujiahui2005@xaas.ac.cn (J.Z.); hzjingy@126.com (J.Y.); wangyf221027@163.com (Y.W.); huiyad0330@163.com (Y.D.); wmjncn@126.com (W.K.); alp68@xaas.ac.cn (A.A.)

**Keywords:** *Gossypium barbadense*, TIM gene family, mitochondrial protein import, allotetraploid cotton, gene expression, fiber development

## Abstract

The translocase of the inner membrane (TIM) family plays an essential role in mediating the transport of nuclear-encoded precursor proteins across the inner mitochondrial membrane and regulating cellular energy metabolism. Sea-island cotton (*Gossypium barbadense*) is valued for its superior fiber quality, yet the composition, evolution, and expression patterns of its *TIM* genes remain unclear. To address this, we performed a genome-wide analysis of the TIM family in *G. barbadense*, using the diploid cotton *G. arboreum*, and *G. raimondii* and the allotetraploid *G. hirsutum* as comparative references. A total of 44 *GbTIM* genes were identified in *G. barbadense* and characterized through phylogenetic, structural, collinearity, promoter, and expression analyses. The TIM family expanded during cotton polyploidization, with tetraploid species containing approximately twice as many genes as diploids. Phylogenetic analysis categorized TIM proteins into seven subfamilies, with Group VI representing the largest clade. Promoter regions were enriched in light- and phytohormone-responsive elements. Interspecific synteny analysis demonstrated extensive collinearity between *G. barbadense* and its diploid ancestors but limited collinearity with *G. hirsutum*, indicating divergence among tetraploids. Transcriptomic profiling revealed highly spatiotemporal expression patterns. *Gbar_A11G001280* was abundantly expressed during fiber initiation and upregulated in low-lint-percentage germplasm, whereas *Gbar_D07G010770* was continuously expressed throughout fiber development. This study clarifies the evolutionary lineage of the TIM family across four cotton species, with a primary focus on *G. barbadense*, and provides candidate genes for molecular breeding of high-quality fiber. Functional validation of these candidate genes is recommended in future studies.

## 1. Introduction

Cotton (*Gossypium* spp.) represents one of the most vital industrial crops globally, serving as the primary raw material for natural textile fibers. During the evolutionary history of the genus *Gossypium*, a pivotal polyploidization event occurred approximately 1.0 to 1.5 million years ago; a diploid ancestor from the A-genome clade (*G. arboreum*) hybridized naturally with a D-genome clade ancestor (*G. raimondii*), followed by chromosome doubling to form two major allotetraploid species, *G. hirsutum* (AD_1_) and *G. barbadense* (AD_2_) [1,2]. Compared with *G. hirsutum* (upland cotton), *G. barbadense* (sea-island or extra-long staple cotton) is renowned for superior fiber quality characterized by exceptional length, strength, and fineness, alongside enhanced resistance to abiotic and biotic stresses [3]. With continuous refinements in high-throughput sequencing and reference genome assemblies across Gossypium taxa, systematically deciphering the evolutionary trajectories and developmental roles of key functional gene families at the genome-wide level has become an indispensable strategy for cotton molecular breeding.

The translocase of the inner membrane (TIM) protein complex serves an imperative function in mitochondrial complex transport and cellular energy metabolism. As essential building blocks of the mitochondrial inner membrane transport system, TIM proteins oversee the translocation, insertion, and assembly of nuclear-encoded precursor proteins synthesized in the cytosol across the inner mitochondrial membrane [4]. Members of this family typically possess conserved Pfam domain architectures such as TIM22, TIM17, or SAM. Beyond maintaining mitochondrial membrane potential and facilitating respiratory chain complex assembly, plant TIM proteins exert multifaceted regulatory influences on abiotic stress adaptation, plant growth (e.g., pollen tube elongation and root morphogenesis), and phytohormone signaling cascades [5,6,7,8,9]. Specifically, the TIM23 translocase complex contains TIM23 and TIM17 as its central transmembrane subunits, which mediate the transport of precursor proteins across the inner mitochondrial membrane [10,11]. Despite sharing structural domain similarities, TIM23 and TIM17 display mechanistically distinct translocation and membrane-integration modes, signifying functional divergence within the family [12]. Furthermore, studies in *Arabidopsis thaliana* have demonstrated that *AtTIM17-1* modulates the timing of seed germination by altering endogenous abscisic acid (ABA) and gibberellin (GA) dynamics [13]. However, impairment of TIM core components compromises mitochondrial DNA import efficiency [14]. These findings highlight that plant TIM proteins not only operate in fundamental protein import, but also actively participate in complex developmental networks through mitochondrial activity regulation.

Nevertheless, systematic characterization, evolutionary dynamics, and expression patterns of the TIM family in allotetraploid cotton species *G. barbadense* remain unexplored, particularly regarding how polyploidization has shaped this gene family and how specific members contribute to fiber development. Polyploidization frequently triggers gene family duplication, functional diversification, and subgenome expression dominance. Genome-wide identification of GbTIM members and systematic profiling of their involvement in fiber development and organogenesis are thus of profound biological relevance. In this study, we conducted a comprehensive genome-wide survey of the TIM family across four representative cotton species (*G. barbadense*, *G. hirsutum*, *G. arboreum*, and *G. raimondii*). We detailed the physicochemical parameters, phylogenetic classification, chromosomal mapping, gene structures, syntenic duplications, and promoter cis-elements of *GbTIM* genes. Utilizing public and tissue-specific transcriptomic datasets, we analyzed *GbTIM* expression dynamics across diverse tissues and fiber developmental stages in extreme lint-percentage sea-island cotton lines. Our findings provide novel insights into the evolutionary expansion of the TIM family in polyploid cotton species and highlight promising candidate loci for fiber quality manipulation.

## 2. Materials and Methods

### 2.1. Identification of TIM Family Members

Whole-genome sequences, coding sequences, and protein annotations for *G. hirsutum* (AD_1__HAU), *G. barbadense* (AD_2__HAU), *G. arboreum* (A_2__HAU), and *G. raimondii* (D_5__HAU) were retrieved from the CottonMD database (https://yanglab.hzau.edu.cn/, accessed on 13 January 2026) [15]. Genomic data for *Arabidopsis thaliana* were downloaded from Ensembl Plants (https://plants.ensembl.org/index.html, accessed on 13 January 2026). A dual-query strategy was deployed to identify TIM family members. Known *Arabidopsis* TIM amino acid sequences extracted from TAIR (https://www.arabidopsis.org, accessed on 10 February 2026) were used as queries for local BLASTp alignment against the cotton protein databases (E-value ≤ 1 × 10^−5^). The Hidden Markov Model (HMM) profile corresponding to the TIM domain (PF02466) was acquired from Pfam and queried against target proteomes using HMMER 3.0 (E-value ≤ 1 × 10^−5^). Candidate genes retrieved from both approaches were intersected. Domain integrity was confirmed using the NCBI Batch CD-Search platform (https://www.ncbi.nlm.nih.gov/Structure/bwrpsb/bwrpsb.cgi, accessed on 3 March 2026) [16]. Genes lacking intact core TIM domains were removed.

### 2.2. Physicochemical Characterization and Subcellular Localization

The physicochemical properties of identified TIM proteins were obtained from the ExPASy database (https://www.expasy.org, accessed on 10 March 2026) [17], including amino acid length, molecular weight (MW), theoretical isoelectric point (pI), instability index, aliphatic index, and grand average of hydropathicity (GRAVY). Subcellular targeting locations were predicted using the WoLF PSORT software program (https://wolfpsort.hgc.jp, accessed on 11 March 2026) [18] under default plant parameters.

### 2.3. Phylogenetic Reconstruction

Multiple sequence alignments of TIM proteins from *A. thaliana* and the four cotton species were generated using ClustalW within MEGA 12 (version 12.1.1). Unrooted phylogenetic trees were constructed using the Maximum Likelihood (ML) method with 1000 bootstrap replicates. The resulting phylogenetic tree was customized using the Interactive Tree Of Life (iTOL) tool (https://itol.embl.de/, accessed on 5 April 2026).

### 2.4. Gene Structure, Conserved Motif, and Domain Architecture Analysis

Exon–intron structures were obtained from the *G. barbadense* genome annotation files. Conserved protein motifs were identified via the MEME suite (http://meme-suite.org/tools/meme, accessed on 15 April 2026), with maximum motif count set to 10 [19]. Conserved functional domains were annotated using the NCBI Conserved Domain Database (CDD, https://ncbiinsights.ncbi.nlm.nih.gov/tag/conserved-domains-database-cdd/, accessed on 15 April 2026). Structural features, conserved motifs, domain layouts, and phylogenetic branching were visualized using TBtools-II (version 2.454) [20].

### 2.5. Chromosomal Mapping

Chromosomal positions and gene densities of *GbTIM* genes were obtained from the genome annotation file. Map diagrams illustrating chromosomal positions were generated using TBtools-II (version 2.454).

### 2.6. Synteny and Duplication Analysis

Intraspecific and interspecific gene collinearity analyses were executed using One Step MCScanX-Super Fast in TBtools-II (version 2.454). Chromosomal distribution and intraspecific duplication events within *G. barbadense* were displayed using Advanced Circos (https://circos.ca/software/download/circos, accessed on 4 May 2026) [21]. Interspecific synteny relationships between *G. barbadense* and the other three cotton species (*G. hirsutum*, *G. arboreum*, and *G. raimondii*) were plotted using Dual Synteny Plot for MCScanX (https://github.com/wyp1125/MCScanX, accessed on 13 May 2026) [22].

### 2.7. Cis-Elements Analysis

The 2000 bp upstream sequence from the transcription start site (TSS) for each GbTIM gene was submitted to the online website PLANTCARE (https://bioinformatics.psb.ugent.be/webtools/plantcare/html, accessed on 18 May 2026) for cis-element prediction [23].

### 2.8. Transcriptomic Profiling and Differential Expression

RNA-seq expression data across root, stem, leaf, petal, anther, bract, filament, pistil, sepal, torus, and different ovule/fiber developmental stages in *G. barbadense* were retrieved from CottonMD. Expression heatmaps were generated with TBtools-II (version 2.454) following row-mean normalization. Transcriptome profiles were further evaluated using two accessions of *G. barbadense* featuring contrasting lint percentages, Ashi (high lint percentage) and Xinhai 33 (XH33, low lint percentage), across multiple fiber development time points (0, 5, 10, 15, 20, 25 DPA).

## 3. Results

### 3.1. Genome-Wide Identification and Physicochemical Traits of Cotton TIM Genes

To provide a comprehensive evolutionary context, we identified TIM family members in representative diploid (*G. arboreum* and *G. raimondii*) and tetraploid (*G. hirsutum* and *G. barbadense*) cotton species, with a total of 22, 22, 40, and 44 members, respectively (Table S1). This demonstrates a two-fold expansion in gene number within tetraploid species compared to their diploid ancestors. Physicochemical evaluations revealed noticeable evolutionary conservation among cotton TIM proteins. Most members exhibited protein lengths ranging between 203 and 207 amino acids, MWs ranging from 21.21 to 21.59 kDa, and weak basic theoretical pI values (7.44–7.76). Instability indices ranged from 47.2 to 47.5, indicating moderate protein instability, while aliphatic index values (76.3–76.9) reflected hydrophobic characteristics. Conversely, GbTIM proteins in *G. barbadense* demonstrated greater structural diversity, with amino acid lengths spanning from 131 to 521 and MWs ranging from 13.6 to 58 kDa. Subcellular predictions suggested that most diploid TIM proteins localize to mitochondria and nuclei, whereas one *GbTIM* member (*Gbar_D11G030130*) in *G. barbadense* was additionally predicted to reside in the cytoplasm (Table S1).

### 3.2. Chromosomal Distribution and Phylogenetic Topology of GbTIMs

**Figure 1:**
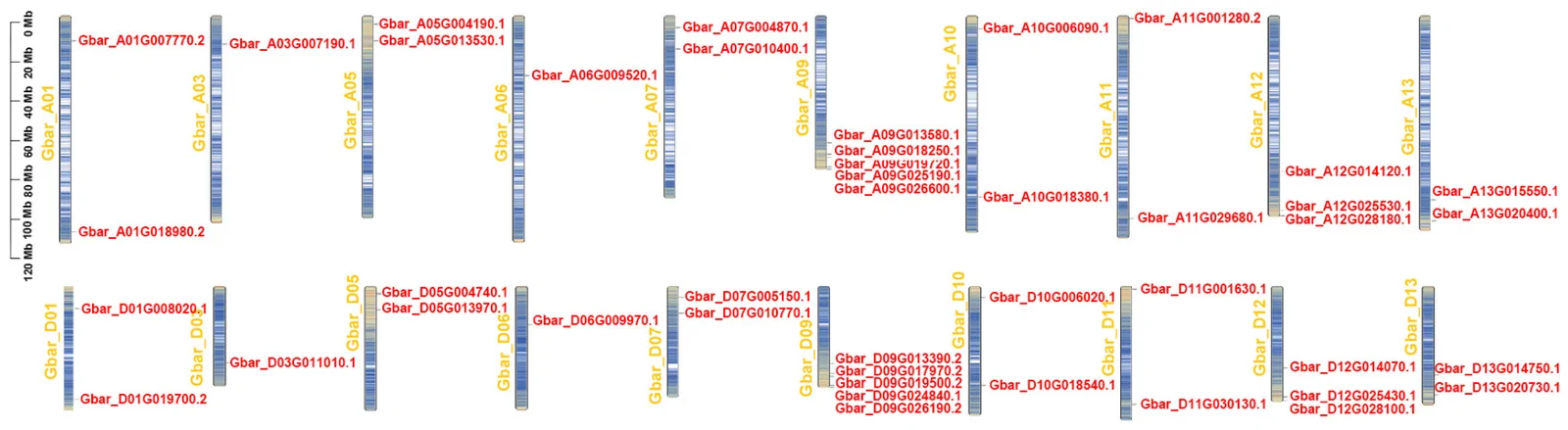
Chromosomal distribution of GbTIM gene family in *G. barbadense*. The scale is in megabases (Mb). Sea-island cotton: *Gossypium barbadense*. Chromosome numbers are highlighted in yellow, and gene IDs are highlighted in red.

**Figure 2:**
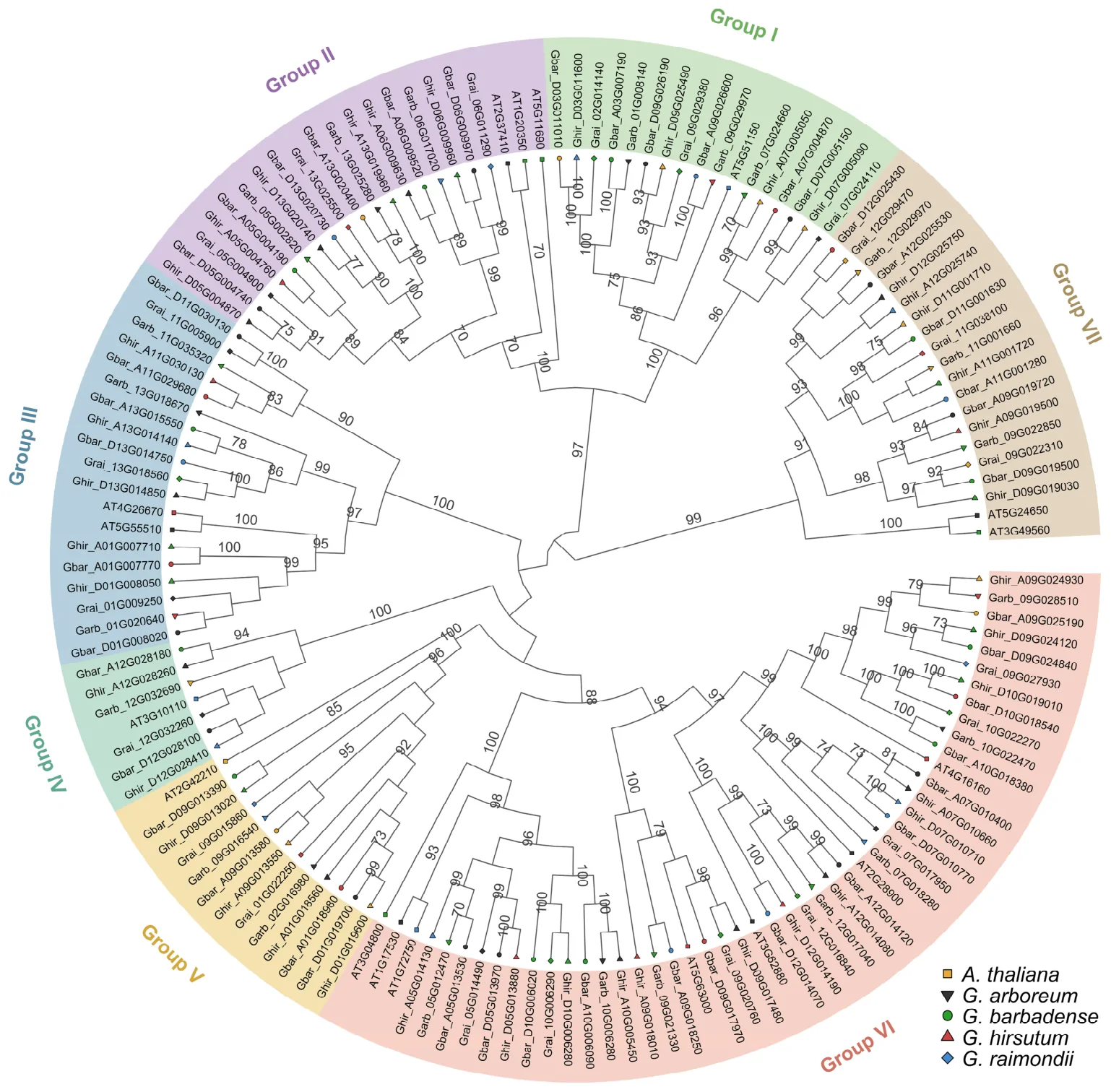
Phylogenetic topology of *TIM* genes in four cotton species and *Arabidopsis*. Arabidopsis: *Arabidopsis thaliana*; upland cotton: *Gossypium hirsutum*; sea-island cotton: *Gossypium barbadense*.

Chromosomal mapping revealed that 44 *GbTIM* genes were broadly distributed across both subgenomes in *G. barbadense* (Figure 1). Chromosomes A02, A04, A08, D02, D04, and D08 contained no GbTIM loci. Gene density clusters were identified on chromosomes A09 and D09, whereas homoeologous chromosome pairs (e.g., A12/D12 and A13/D13) harbored symmetrical gene distributions. A maximum likelihood phylogenetic tree was constructed using 146 TIM amino acid sequences from *A. thaliana* and four cotton species (Figure 2). The members were divided into seven evolutionary subfamilies (Group I–VII), containing 18, 21, 19, 7, 13, 48, and 20 members, respectively. Among the *G. barbadense* members, Group VI contained the largest gene complement, whereas Group IV was the smallest, reflecting lineage-specific expansion rates during polyploid evolution.

### 3.3. Gene Architecture and Domain Compositions

**Figure 3:**
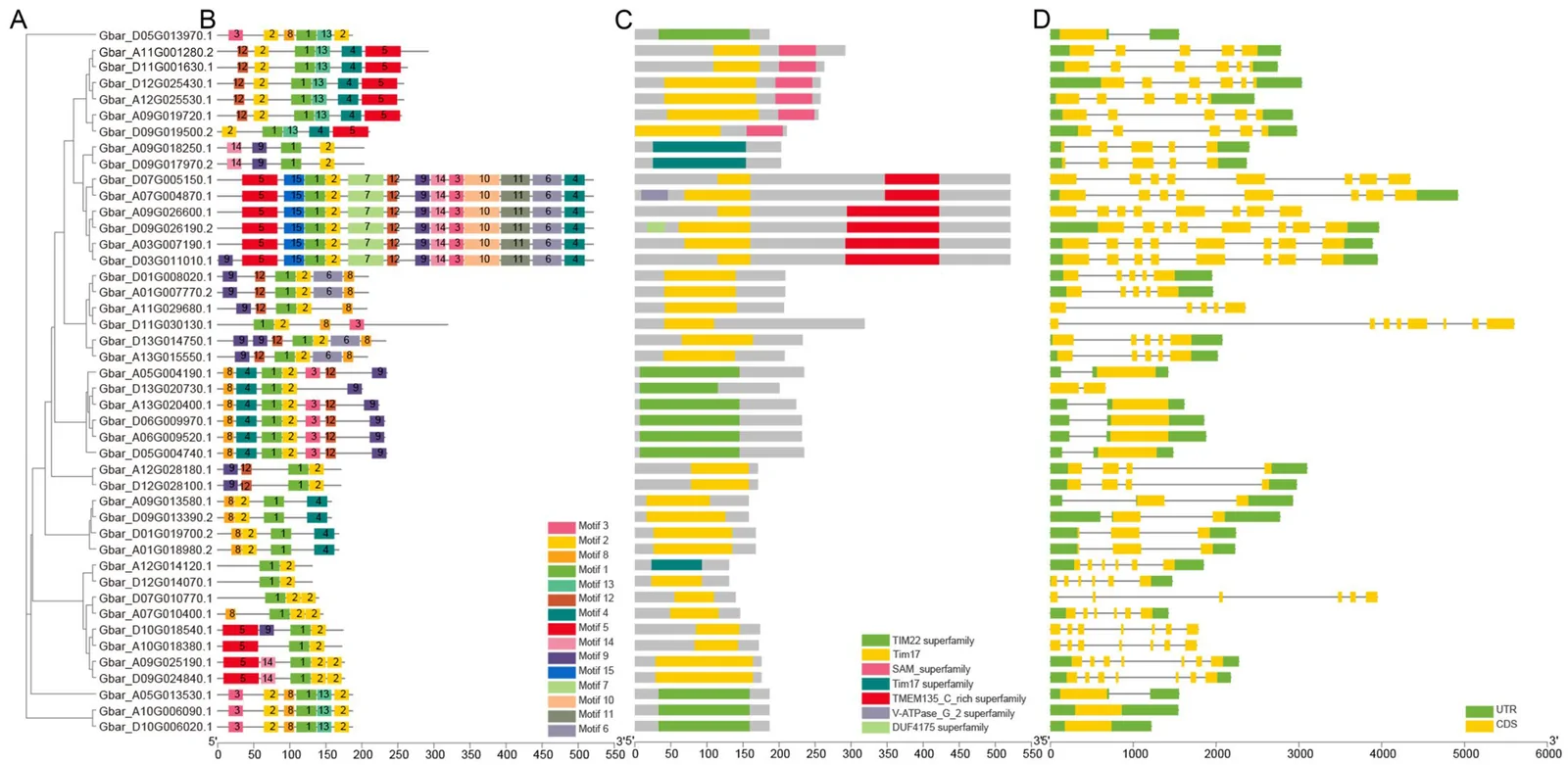
Evolutionary relationships (**A**), conserved motifs (**B**), domain compositions (**C**), and gene structures (**D**) of the GbTIM gene family in *G. barbadense*. Sea-island cotton: *Gossypium barbadense*.

To evaluate structural diversification, conserved motifs, Pfam domains, and exon-intron architectures of *GbTIM* genes were integrated with the phylogenetic layout. Conserved motifs were shared among closely related members within the same clade (Figure 3A,B). Motifs 1 and 2 were present across all 44 GbTIM proteins. Domain annotations confirmed that GbTIM proteins harbor canonical TIM22, TIM17, or SAM superfamily domains (Figure 3C). Exon–intron configurations revealed structural diversity, and exon counts ranged from 1 to 8, with intronless genes observed in specific clades (Figure 3D).

### 3.4. Intraspecific and Interspecific Synteny of GbTIMs

**Figure 4:**
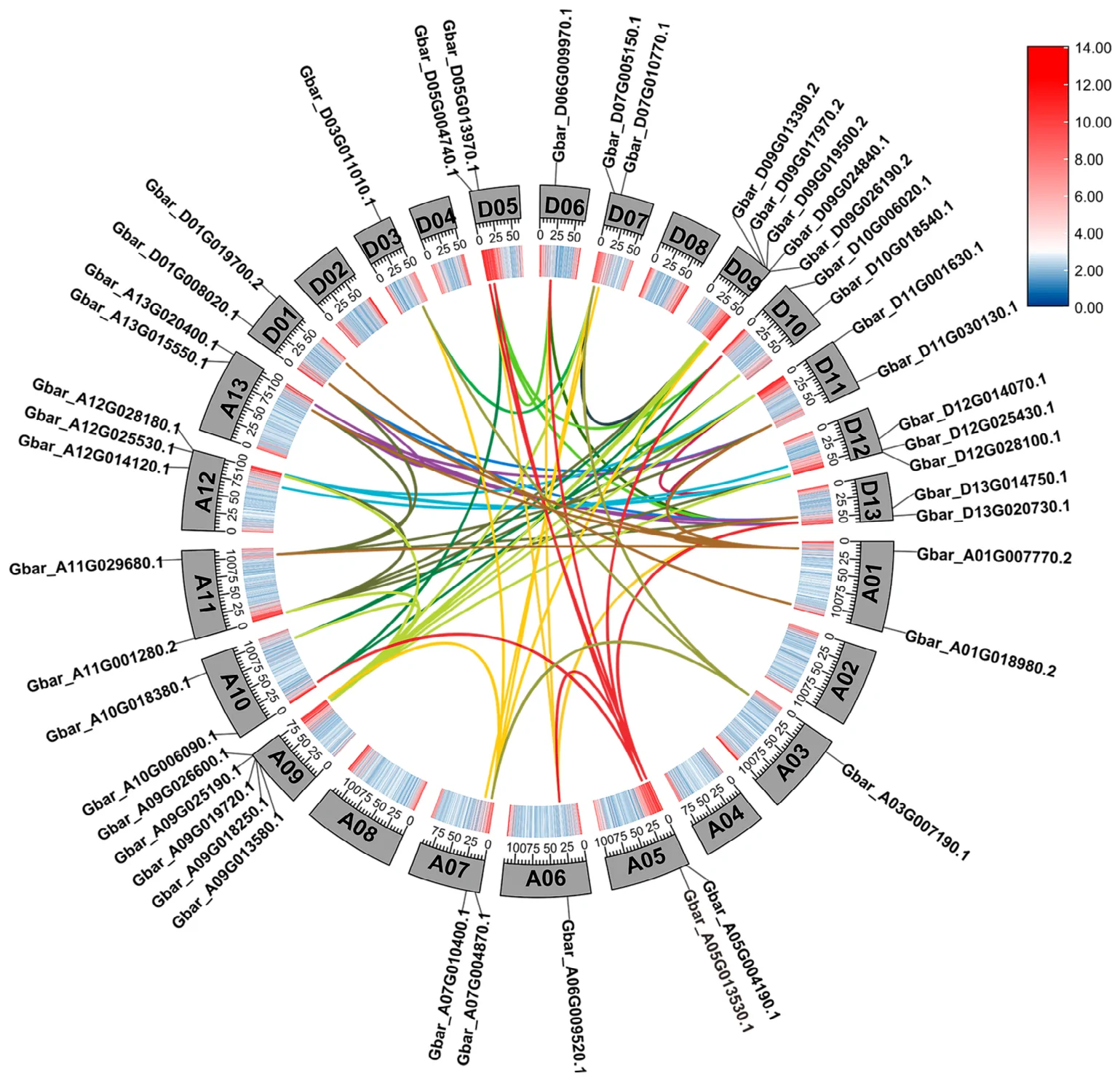
Intraspecific synteny of the GbTIM gene family in *G. barbadense*. The outer circle represents the chromosomes (A01–A13 and D01–D13). The red-blue heatmap (scale 0.00–14.00) indicates gene density across the chromosomes. The inner colored lines represent intra-genomic collinear blocks; different colors are used to visually distinguish different syntenic regions. Sea-island cotton: *Gossypium barbadense*.

**Figure 5:**
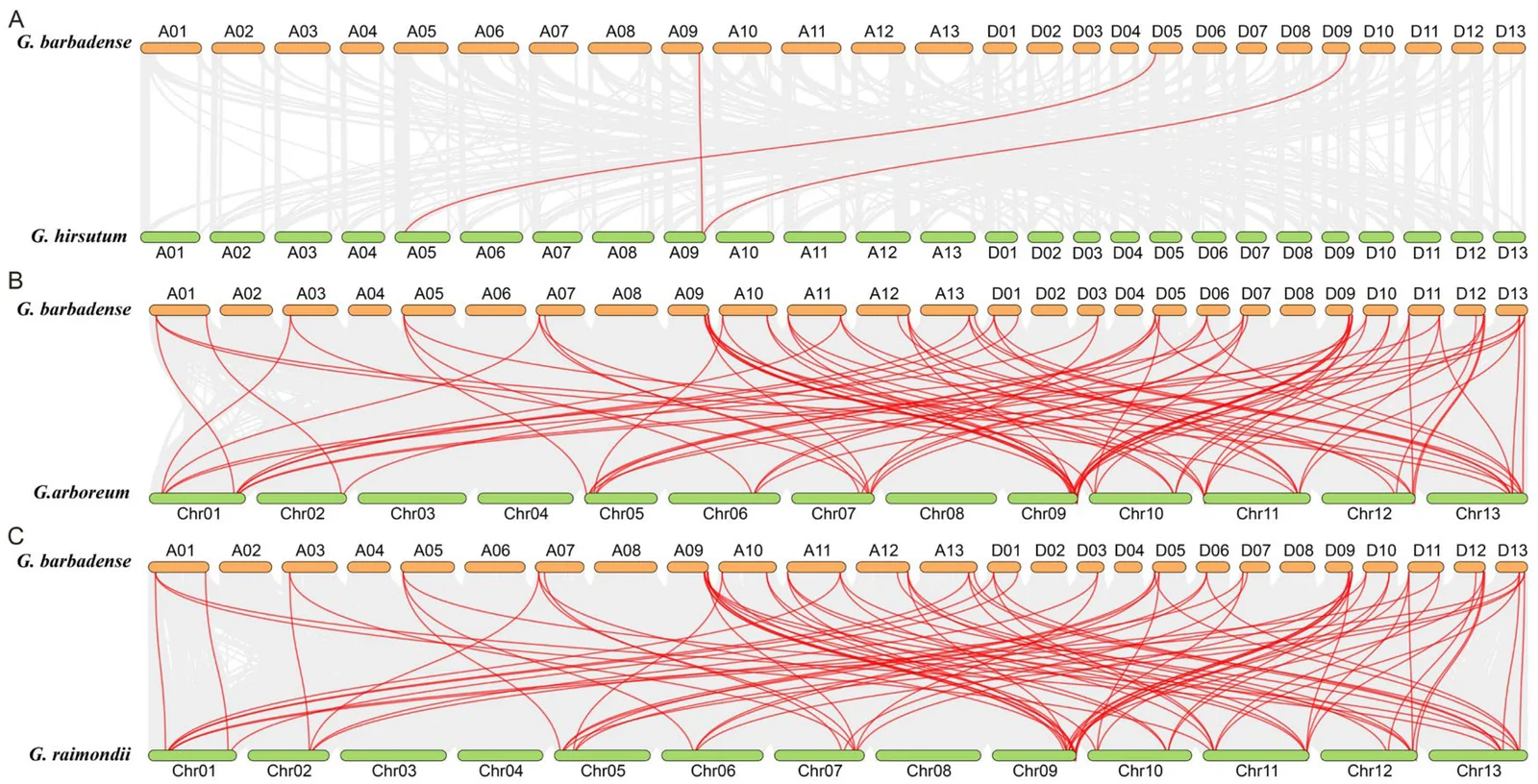
Interspecific synteny of the GbTIM gene family with *G. hirsutum* (**A**), *G. arboreum* (**B**), *G. raimondii* (**C**). Upland cotton: *Gossypium hirsutum*; sea-island cotton: *Gossypium barbadense*. Red lines highlighted the duplicated *TIM* gene pairs, and gray lines in the background indicated the collinear blocks.

Intraspecific synteny analysis identified 67 collinear gene pairs within the *G. barbadense* genome (Figure 4). These included 11 segmental duplication pairs within the At subgenome, 12 within the Dt subgenome, and 44 inter-subgenomic duplication pairs between At and Dt. Segmental duplication across subgenomes served as the primary mechanism driving GbTIM family expansion. Interspecific synteny analysis revealed that *G. barbadense* maintained 84 collinear pairs with diploid *G. arboreum* (A_2_) and 82 pairs with *G. raimondii* (D_5_) (Figure 5). In contrast, *G. barbadense* shared only 3 syntenic pairs with allotetraploid *G. hirsutum* (AD_1_). This indicates that while ancestral genomic synteny was preserved following polyploidization, extensive lineage-specific genomic rearrangements or gene loss occurred after the divergence of the two tetraploid species.

### 3.5. Promoter Cis-Regulatory Element Analysis

**Figure 6:**
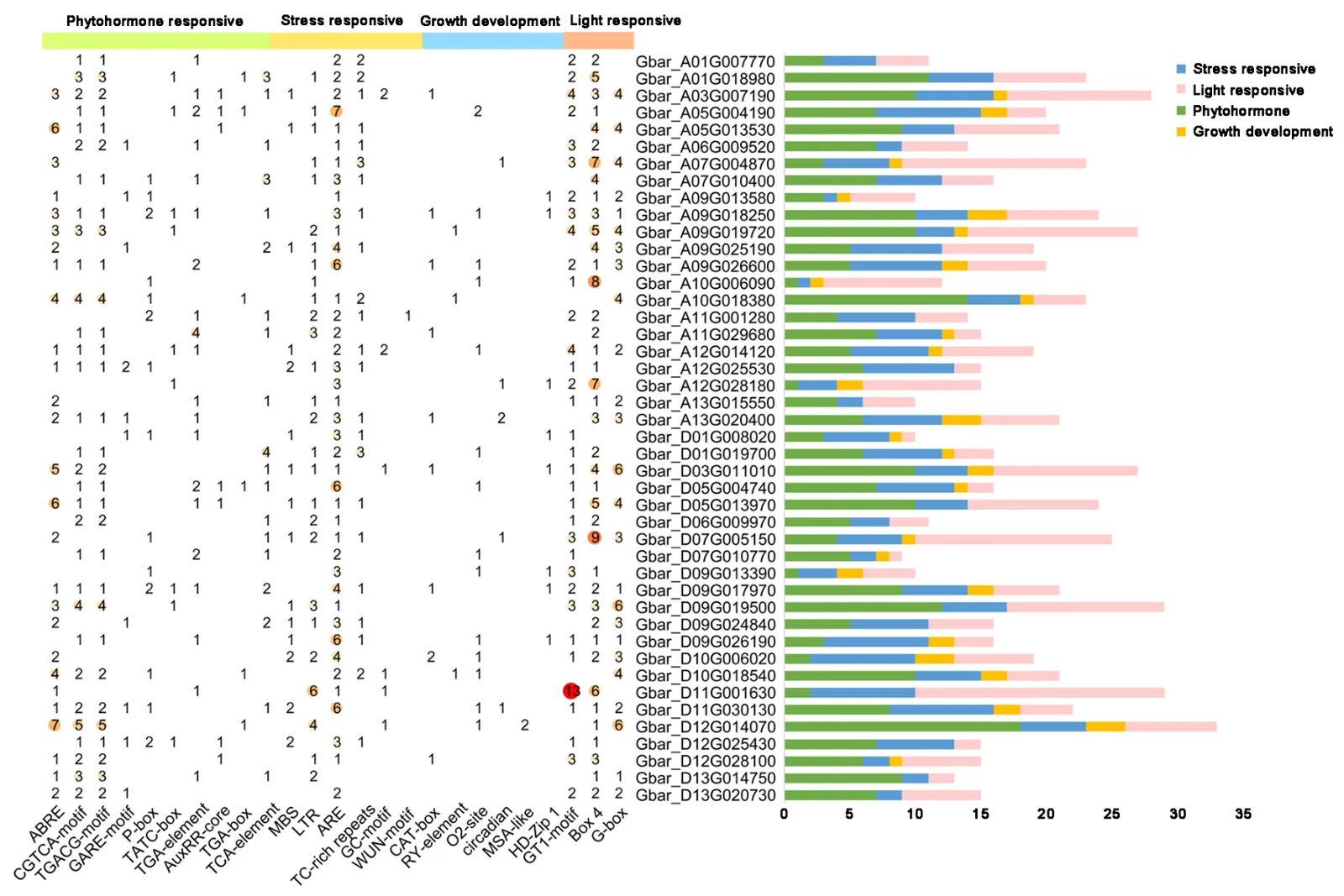
Cis-acting elements existed in promoters of *GbTIM* genes. Sea-island cotton: *Gossypium barbadense*.

Scan of the 2.0 kb upstream promoter regions of 44 *GbTIM* genes identified various cis-regulatory elements, including light-responsive elements (e.g., Box4 and G-box), phytohormone elements (ABRE for ABA-responsive, CGTCA/TGACG motifs for MeJA, and P-box for gibberellin), and stress-responsive elements (MBS for drought and ARE for anaerobic induction) (Figure 6). *Gbar_D12G014070* harbored the highest density of light-responsive elements. Notably, these in silico predictions indicate potential regulatory interactions, but the actual functionality of these motifs and their cell-type-specific activities require experimental validation.

### 3.6. Spatiotemporal Expression Dynamics and Fiber Development Profiles

**Figure 7:**
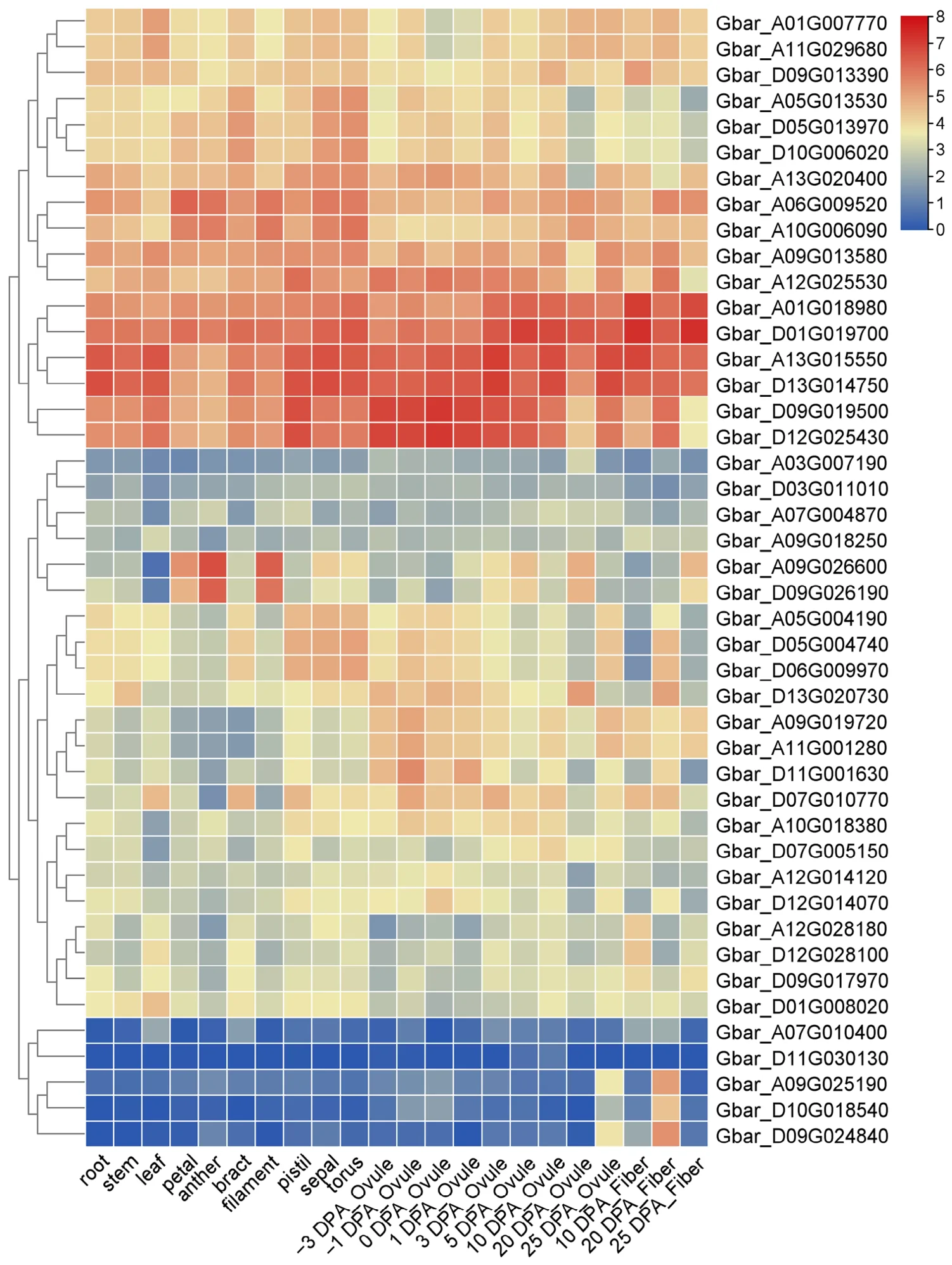
Expression profiles of *GbTIM*s across different tissues and developmental stages. Transcriptomic data were row-mean normalized, with red intensity representing the level of gene expression. The redder the color, the higher the gene expression level. Sea-island cotton: *Gossypium barbadense*.

**Figure 8:**
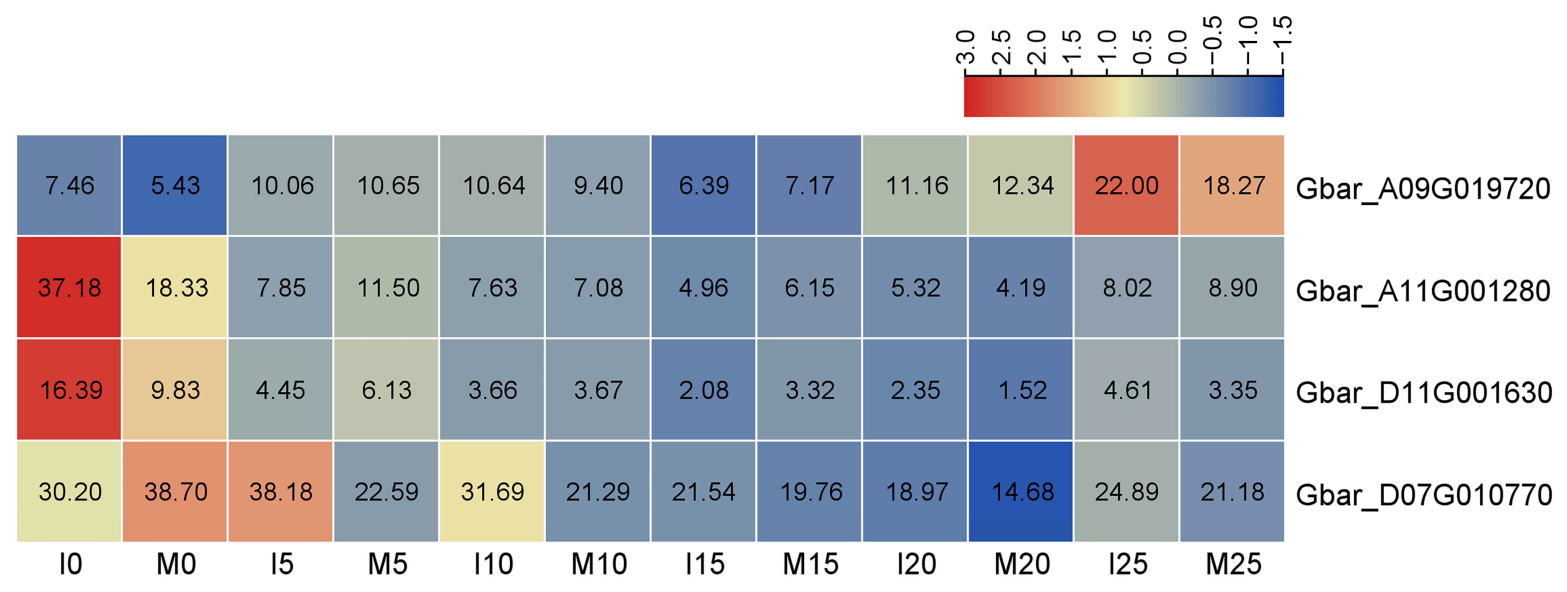
The expression of *GbTIM*s in extreme lint-percentage cotton materials. I: low-lint-percentage sea-island cotton acc. XH33; M: high-lint-percentage sea-island cotton acc. Ashi. 0, 5, 10, 15, 20, 25 represent samples collected at 0, 5, 10, 15, 20, 25 DPA. Sea-island cotton: *Gossypium barbadense*.

Transcriptomic profiling at the tissue level revealed expression divergence among *GbTIM* genes across different organs and developmental stages (Figure 7). A core module comprising 17 genes (including *Gbar_A01G018980* and *Gbar_D01G019700*) displayed relatively ubiquitous expression across all tissues examined, suggesting potential roles in fundamental metabolic processes. Conversely, *Gbar_A09G026600* and *Gbar_D09G026190* displayed preferential expression in floral tissues (petals, anthers, and filaments). Five genes, including *Gbar_D11G030130,* showed minimal transcript abundance across all samples tested. Temporal expression patterns were also observed during ovule development; for example, *Gbar_A09G019720* and *Gbar_A11G001280* exhibited higher transcript levels at early stages (−3 to 1 DPA) compared to later stages (10 to 25 DPA). However, as these data were derived from whole-tissue RNA-seq, the expression patterns represent average signals from mixed cell populations. Further studies at single-cell resolution or via in situ hybridization are needed to determine whether these genes are expressed uniformly within a tissue or restricted to specific cell types. Moreover, analysis of extreme lint-percentage lines revealed that *Gbar_D07G010770* maintained high transcript levels throughout fiber elongation. In contrast, *Gbar_A11G001280* was specifically upregulated during fiber initiation (0 DPA), with significantly higher expression in the low-lint-percentage accession (XH33) than in the high-lint-percentage line (Ashi) (Figure 8).

## 4. Discussion

Mitochondrial homeostasis relies on the translocation of cytosolically synthesized preproteins through inner membrane translocase complexes. TIM23 and TIM22 complexes operate as primary entry channels, governing the targeting and assembly of metabolic enzymes and respiratory chain components [24,25]. In *Arabidopsis,* TIM proteins regulate mitochondrial biogenesis, influencing photosynthetic efficiency, anthocyanin accumulation, hormone crosstalk, and seed development [9,26]. However, the evolutionary dynamics and functional roles of the TIM family in allotetraploid sea-island cotton had not been systematically characterized. Here, we identified 44 *GbTIM* genes in *G. barbadense*, providing insights into the evolution and functional specialization of mitochondrial transport systems in polyploid cotton.

Genome-wide identification offers a framework for understanding complex trait evolution in polyploid species. Allotetraploid cotton evolution involved whole-genome duplication and extensive segmental duplication [27,28,29,30,31]. Our results show that the TIM family doubled in size in tetraploid cottons relative to diploid ancestors. Subcellular targeting predictions consistently indicated mitochondrial localization, highlighting functional conservation. Structural variations, including exon number divergence and intronless members, point to potential alternative splicing or pseudogenization events during polyploid evolution [32].

Phylogenetic reconstruction divided cotton TIM proteins into seven subfamilies, with Group VI and IV representing the largest and smallest clades. The number of *TIM* genes in the *Gossypium* genus is much higher than that in *Arabidopsis*, indicating that this family has undergone significant species-specific expansion during the evolution of the *Gossypium* genus. This is consistent with the differentiation pattern of the TIM family sub-members in early eukaryotes, suggesting that its core function is strongly conserved during plant evolution [11]. Members within the same subgroup are highly conserved in gene structure, while there are significant differences between different subgroups. This feature has also been observed in gene families such as ERF, GAox, and cellulose synthase [33,34,35], suggesting an intrinsic relationship between structural differentiation and the formation of functional diversity. Chromosomal distribution demonstrated gene enrichment on homoeologous chromosomes A09 and D09, indicating that localized duplication contributed to family expansion.

Synteny analysis further showed that inter-subgenomic (At-Dt) duplication exceeded intra-subgenomic events, identifying cross-subgenome duplications as the primary driver of expansion. Notably, *G. barbadense* maintained high synteny with diploid ancestors but showed significant syntenic divergence from allotetraploid *G. hirsutum*. This indicates that despite shared ancestry, independent polyploid lineages underwent differential gene loss, structural rearrangements, and copy number variations, consistent with evolutionary patterns reported for AGP and Trihelix families [28,36,37,38].

*Arabidopsis* TIM17 and TIM23 subtypes have shown temporal and spatial specificity, and the general and carrier import pathways may be differently regulated [10]. Here, expression profiling provided evidence of functional specialization among *GbTIM* genes. While constitutively active members maintain basic metabolic functions, others exhibited organ- or stage-specific expression. Selective expression in floral organs and early ovules (−3 to 1 DPA), a critical window determining fiber cell initiation density [39,40], suggests involvement in early cell differentiation. Enriched light- and hormone-responsive promoter elements suggest that GbTIM proteins may integrate environmental and hormonal signals with mitochondrial energy supply during fiber initiation. Differential expression of *Gbar_A11G001280* in contrasting lint-percentage lines highlights its potential utility as a target for modulating fiber cell initiation.

## 5. Conclusions

This study systematically characterized the TIM gene family in allotetraploid *G. barbadense*, identifying 44 *GbTIM* genes and demonstrating that inter-subgenomic segmental duplication served as the primary mechanism underlying their expansion. Comparative synteny analyses across four cotton species revealed conserved collinearity between *G. barbadense* and its diploid progenitors, while limited synteny with *G. hirsutum* indicated substantial genomic divergence between tetraploid cottons. Expression profiling further identified candidate genes, including *Gbar_A09G019720* and *Gbar_A11G001280*, with preferential expression in floral organs or during fiber initiation, suggesting their potential roles in fiber quality formation. Collectively, these findings not only advance our understanding of mitochondrial translocase evolution in polyploid cotton but also provide promising target loci for molecular breeding aimed at fiber improvement. Given that the current conclusions are derived primarily from genomic and transcriptomic analyses, future functional validation using transgenic or gene-editing approaches will be essential to confirm the biological functions of these candidate genes.

## Data Availability

The original data presented in the study are openly available in CottonMD at https://yanglab.hzau.edu.cn/CottonMD, accessed on 13 January 2026. The fiber development transcriptome data for extreme lint-percentage lines (Ashi and XH33) are openly available in the Gene Expression Omnibus (GEO) repository under accession number GSE184965.

## Supplementary Materials

The following supporting information can be downloaded at https://www.mdpi.com/article/10.3390/cimb48090929/s1.
