# Supplementary material for: Genome-Wide Identification and Expression Analysis of the TIM Gene Family in Sea-Island Cotton (Gossypium barbadense) During Fiber Development
Source: Curr Issues Mol Biol. 2026 Sep 11;48(9):929. doi: 10.3390/cimb48090929 (PMC13605187; doi:10.3390/cimb48090929)
Supplement: Supplementary file 1 [file cimb-48-00929-s001.zip › cimb-4517035-supplementary.pdf]

Table S1. Physicochemical properties and subcellular localization of TIM gene family members in four cotton species.

Gene ID	Number of amino acid	Molecular weight (Da)	Theoretical pI	Instability index	Aliphatic index	Hydropathicity	Localization
Garb_01G008140	521	58012.28	9.48	52.7	94.41	-0.052	Mitochondrion
Garb_01G020640	209	21688.61	8.2	40.73	78.95	0.029	Mitochondrion
Garb_02G016980	160	17156.61	9.12	24.54	78.62	-0.006	Mitochondrion
Garb_05G002820	235	24280.43	5.44	59.3	64.43	-0.068	Mitochondrion
Garb_05G012470	187	19869.53	9.51	33.47	93.42	-0.044	Mitochondrion
Garb_06G017020	232	24174.43	5.86	64.25	64.83	-0.158	Mitochondrion
Garb_07G018280	146	15462.74	9.2	10.37	90.89	0.102	Mitochondrion
Garb_07G024660	521	57929.13	9.4	50.59	92.34	-0.077	Mitochondrion
Garb_09G016540	158	16900.36	9.48	27.74	77.22	-0.006	Mitochondrion
Garb_09G021330	203	21695.95	9.27	23.02	88.47	-0.058	Mitochondrion
Garb_09G022850	247	26411.5	9.55	36.7	86.92	0.032	Mitochondrion
Garb_09G028510	176	18701.32	8.91	22.98	83.3	-0.147	Mitochondrion
Garb_09G029970	521	57829.96	9.43	53.63	94.05	-0.054	Mitochondrion
Garb_10G006280	187	19790.32	9.16	35.87	90.27	-0.063	Mitochondrion
Garb_10G022470	172	18161.5	6.51	26.29	79.59	-0.101	Mitochondrion
Garb_11G001660	263	28136.35	9.6	43.82	81.25	-0.184	Mitochondrion
Garb_11G035320	207	21639.92	9.28	47.14	85.36	0.082	Plastid
Garb_12G017040	131	13602.64	9.55	40.13	90.99	0.399	Mitochondrion
Garb_12G029970	258	27617.78	9.64	43.84	83.64	-0.159	Mitochondrion
Garb_12G032690	171	18180.86	6.82	47.93	67.89	0.007	Mitochondrion
Garb_13G018670	208	21566.62	9.08	39.96	76.11	0.032	Plastid
Garb_13G025280	224	23149.35	5.2	59.2	70.62	-0.008	Mitochondrion
Grai_01G009250	209	21688.61	8.2	40.73	78.95	0.029	Mitochondrion
Grai_01G022250	160	17128.56	9.12	24.95	77.44	-0.021	Mitochondrion
Grai_02G014140	521	58005.27	9.48	50.52	95.36	-0.049	Mitochondrion
Grai_05G004900	235	24260.4	5.44	60.3	65.66	-0.068	Mitochondrion
Grai_05G014490	187	19812.44	9.33	30.59	91.82	-0.055	Mitochondrion

Grai_06G011290	232	24163.37	5.68	62.85	63.58	-0.159	Mitochondrion
Grai_07G017950	146	15434.69	9.2	10.95	89.59	0.086	Mitochondrion
Grai_07G024110	977	108411.44	9.07	44.52	91.18	-0.019	Cell membrane
Grai_09G015860	158	16882.3	9.33	27.94	77.78	-0.033	Mitochondrion
Grai_09G020760	203	21653.86	9.27	23.02	87.04	-0.08	Mitochondrion
Grai_09G022310	255	27143.38	9.35	35.18	84.59	0.027	Mitochondrion
Grai_09G027930	176	18654.31	8.91	22.75	83.86	-0.122	Mitochondrion
Grai_09G029380	540	60047.17	9.22	50.86	91.46	-0.113	Mitochondrion
Grai_10G006290	187	19796.32	9.13	36.32	88.18	-0.065	Mitochondrion
Grai_10G022270	172	18180.49	6.28	30.34	84.13	-0.126	Mitochondrion
Grai_11G005900	146	15376.93	9.57	48.82	91.58	0.113	Mitochondrion
Grai_11G038100	263	28097.3	9.75	43.27	84.22	-0.173	Mitochondrion
Grai_12G016840	131	13735.81	9.55	41.22	87.25	0.364	Mitochondrion
Grai_12G029470	258	27669.85	9.64	45.26	82.87	-0.185	Mitochondrion
Grai_12G032260	171	18229.04	7.64	49.06	68.48	-0.003	Mitochondrion
Grai_13G018560	233	24387.98	9.61	41.76	72.53	-0.075	Plastid
Grai_13G025500	224	23140.24	5.2	54.7	69.33	-0.032	Mitochondrion
Gbar_A01G007770.2	209	21715.63	8.53	41.91	78.95	0.026	Mitochondrion
Gbar_A01G018980.2	168	18237.93	9.16	25.94	74.88	0.018	Mitochondrion
Gbar_A03G007190.1	521	58012.28	9.48	52.7	94.41	-0.052	Mitochondrion
Gbar_A05G004190.1	235	24220.33	5.44	60.94	64.43	-0.083	Mitochondrion
Gbar_A05G013530.1	187	19903.55	9.51	33.47	91.34	-0.049	Mitochondrion
Gbar_A06G009520.1	232	24164.39	5.86	65.32	64.83	-0.154	Mitochondrion
Gbar_A07G004870.1	521	58043.32	9.4	50.01	92.51	-0.072	Mitochondrion
Gbar_A07G010400.1	146	15425.68	9.2	10.43	89.59	0.084	Mitochondrion
Gbar_A09G013580.1	158	16886.33	9.48	27.8	76.58	-0.008	Mitochondrion
Gbar_A09G018250.1	203	21695.95	9.27	23.02	88.47	-0.058	Mitochondrion
Gbar_A09G019720.1	255	27164.36	8.99	35.82	84.2	0.027	Mitochondrion
Gbar_A09G025190.1	176	18701.32	8.91	22.98	83.3	-0.147	Mitochondrion

Gbar_A09G026600.1	521	57829.96	9.43	53.63	94.05	-0.054	Mitochondrion
Gbar_A10G006090.1	187	19790.32	9.16	35.87	90.27	-0.063	Mitochondrion
Gbar_A10G018380.1	172	18128.43	6.28	27.16	79.59	-0.094	Mitochondrion
Gbar_A11G001280.2	292	31519.32	9.62	42.68	85.55	-0.11	Mitochondrion
Gbar_A11G029680.1	207	21588.81	9.01	47	83.96	0.086	Plastid
Gbar_A12G014120.1	131	13616.67	9.55	40.13	91.76	0.402	Mitochondrion
Gbar_A12G025530.1	258	27642.8	9.52	42.92	81.74	-0.177	Mitochondrion
Gbar_A12G028180.1	171	18166.84	6.82	47.93	67.89	0.007	Mitochondrion
Gbar_A13G015550.1	208	21541.63	9.14	41.59	76.11	0.024	Plastid
Gbar_A13G020400.1	224	23135.32	5.2	57.48	70.18	-0.009	Mitochondrion
Gbar_D01G008020.1	209	21688.61	8.2	40.73	78.95	0.029	Mitochondrion
Gbar_D01G019700.2	168	18223.95	9.3	26.33	73.75	0.002	Mitochondrion
Gbar_D03G011010.1	521	58024.34	9.46	51.8	95.91	-0.027	Mitochondrion
Gbar_D05G004740.1	235	24234.32	5.44	61.12	64	-0.087	Mitochondrion
Gbar_D05G013970.1	187	19812.43	9.1	30.19	92.83	-0.037	Mitochondrion
Gbar_D06G009970.1	232	24177.4	5.69	63.68	63.58	-0.159	Mitochondrion
Gbar_D07G005150.1	521	58035.27	9.34	51.62	91.98	-0.072	Mitochondrion
Gbar_D07G010770.1	140	15504.24	8.67	17.23	110.71	0.334	Mitochondrion
Gbar_D09G013390.2	158	16941.37	9.48	29.03	77.78	-0.051	Mitochondrion
Gbar_D09G017970.2	203	21653.86	9.27	23.02	87.04	-0.08	Mitochondrion
Gbar_D09G019500.2	211	22206.59	9.37	36.08	85.59	0.158	Mitochondrion
Gbar_D09G024840.1	176	18644.27	8.91	24.7	83.86	-0.118	Mitochondrion
Gbar_D09G026190.2	521	57941.07	9.39	51.9	93.86	-0.06	Mitochondrion
Gbar_D10G006020.1	187	19796.32	9.13	36.32	88.18	-0.065	Mitochondrion
Gbar_D10G018540.1	174	18464.85	7.01	31.98	83.16	-0.173	Mitochondrion
Gbar_D11G001630.1	263	28111.37	9.81	43.85	84.22	-0.175	Mitochondrion
Gbar_D11G030130.1	319	35059.28	9.45	44.22	83.17	-0.294	Cytoplasm
Gbar_D12G014070.1	131	13707.69	9.55	40.28	85.04	0.302	Mitochondrion
Gbar_D12G025430.1	258	27669.85	9.64	45.26	82.87	-0.185	Mitochondrion

Gbar_D12G028100.1	171	18214.97	6.82	49.06	68.48	-0.001	Mitochondrion
Gbar_D13G014750.1	233	24387.98	9.61	41.76	72.53	-0.075	Plastid
Gbar_D13G020730.1	201	20927.18	5.85	55.04	56.87	-0.346	Mitochondrion
Ghir_A01G007710.1	209	21716.62	8.21	42.83	78.95	0.026	Mitochondrion
Ghir_A01G018560.1	160	17156.61	9.12	24.54	78.62	-0.006	Mitochondrion
Ghir_A05G004760.1	235	24220.33	5.44	60.94	64.43	-0.083	Mitochondrion
Ghir_A05G014130.1	191	20464.23	8.69	36.4	89.42	-0.132	Mitochondrion
Ghir_A06G009630.1	232	24164.39	5.86	65.32	64.83	-0.154	Mitochondrion
Ghir_A07G005050.1	521	57989.22	9.4	49.93	92.34	-0.07	Mitochondrion
Ghir_A07G010660.1	146	15441.68	9.17	9.85	89.59	0.055	Mitochondrion
Ghir_A09G013550.1	158	16886.33	9.48	27.8	76.58	-0.008	Mitochondrion
Ghir_A09G018010.1	192	20426.39	7.87	23.4	85.94	-0.099	Mitochondrion
Ghir_A09G019500.1	211	22273.69	9.56	36.99	85.12	0.12	Mitochondrion
Ghir_A09G024930.1	176	18701.32	8.91	22.98	83.3	-0.147	Mitochondrion
Ghir_A10G005450.1	187	19790.32	9.16	35.87	90.27	-0.063	Mitochondrion
Ghir_A11G001720.1	240	26268.42	9.96	43.88	67.5	-0.245	Mitochondrion
Ghir_A11G030130.1	207	21600.82	9.01	48.34	83	0.069	Plastid
Ghir_A12G014080.1	132	13788.9	9.76	39.33	94.02	0.405	Mitochondrion
Ghir_A12G025740.1	243	25938.87	9.52	41.78	80.78	-0.131	Mitochondrion
Ghir_A12G028260.1	171	18166.84	6.82	47.93	67.89	0.007	Mitochondrion
Ghir_A13G014140.1	208	21585.67	9.22	40.66	76.11	0.025	Plastid
Ghir_A13G019960.1	208	21771.07	5.83	63.11	83.94	0.203	Mitochondrion
Ghir_D01G008050.1	209	21716.66	8.2	41.13	79.86	0.041	Mitochondrion
Ghir_D01G019600.2	168	18223.95	9.3	26.33	73.75	0.002	Mitochondrion
Ghir_D03G011600.1	521	57965.27	9.42	51.94	95.91	-0.022	Mitochondrion
Ghir_D05G004870.1	235	24234.32	5.44	61.12	64	-0.087	Mitochondrion
Ghir_D05G013880.1	187	19812.43	9.1	30.19	92.83	-0.037	Mitochondrion
Ghir_D06G009960.1	232	24177.4	5.69	63.68	63.58	-0.159	Mitochondrion
Ghir_D07G005090.1	521	58035.31	9.37	51.17	91.98	-0.073	Mitochondrion
Ghir_D07G010710.1	140	15414.14	9.01	17.16	113.5	0.374	Mitochondrion

Ghir_D09G013020.1	158	16913.35	9.45	32.16	77.78	-0.047	Mitochondrion
Ghir_D09G017480.1	203	21653.86	9.27	23.02	87.04	-0.08	Mitochondrion
Ghir_D09G019030.1	255	27129.31	9.21	34.93	84.59	0.029	Mitochondrion
Ghir_D09G024120.1	176	18644.27	8.91	24.7	83.86	-0.118	Mitochondrion
Ghir_D09G025490.1	524	58343.43	9.31	51.59	93.51	-0.068	Mitochondrion
Ghir_D10G006280.2	187	19796.32	9.13	36.32	88.18	-0.065	Mitochondrion
Ghir_D10G019010.1	174	18506.93	7.01	33.09	84.83	-0.147	Mitochondrion
Ghir_D11G001710.1	263	28111.37	9.81	43.85	84.22	-0.175	Mitochondrion
Ghir_D12G014190.1	131	13707.69	9.55	40.28	85.04	0.302	Mitochondrion
Ghir_D12G025750.1	246	26419.44	9.85	45.76	78.98	-0.199	Mitochondrion
Ghir_D12G028410.1	171	18214.97	6.82	49.06	68.48	-0.001	Mitochondrion
Ghir_D13G014850.1	233	24387.98	9.61	41.76	72.53	-0.075	Plastid
Ghir_D13G020740.1	224	23200.35	6.58	55.25	66.29	-0.032	Mitochondrion
